# Foveal hyper-reflective vertical lines detected by optical coherence tomography: Imaging features, literature review and differential diagnoses

**DOI:** 10.1007/s00417-024-06616-5

**Published:** 2024-10-15

**Authors:** Adi Porat Rein, Hashem Totah, Koby Brosh, David Zadok, Joel Hanhart

**Affiliations:** Department of Ophthalmology, Shaare Zedek Medical Center, affiliated to the Hebrew University, Shmuel Bait St 12, 9103102 Jerusalem, Israel

**Keywords:** Fovea, Hyper-reflectivity, Macular edema, OCT

## Abstract

**Purpose:**

To describe foveal hyper-reflective vertical lines (FVL) as a specific morphological finding on structural spectral-domain optical coherence tomography (SD-OCT) and discuss its differential diagnosis.

**Methods:**

Observational case series. Ten patients (10 eyes) with FVL were meticulously examined at the Ophthalmology Department, Shaare Zedek Medical Center, Jerusalem, Israel. Detailed analysis of SD-OCT findings, clinical records, and retinal imaging was conducted to establish correlations between FVL and various underlying conditions.

**Results:**

We established the following list of settings, supported by the clinical context and ancillary investigations, in which SD-OCT displayed FVL: inflammation (1 eye), mechanical (1 eye), resorption of fluids of various origins (4 eyes), macular telangiectasia (1 eye), age-related macular degeneration (1 eye), diabetic retinopathy (1 eye) and scar (1 eye).

**Conclusions:**

FVL can be observed in various underlying conditions. Recognition of this pattern and formulation of an appropriate differential diagnosis is of interest for correctly diagnosing and treating patients whose structural OCT harbors this yet overlooked finding.

## Introduction

Optical coherence tomography (OCT) is a non-invasive imaging technique that enables precise visualization of the retinal layers. In several clinical contexts, foveal hyper-reflective vertical lines (FVL) are observed at the fovea.

The appearance of FVL after the resolution of macular edema was termed 'track lines' by Hasegawa et al. [1] Scharf et al., as well as Ishibashi et al., coined 'hyperreflective stress lines', also called 'foveal crack sign', as an early marker of full-thickness macular holes (FTMH) and lamellar macular holes (LMH) development. [2, 3] Cohen and his collaborators, who conducted an extensive study on this peculiar pattern, proposed the term 'intraretinal hyperreflective lines' [4–6].

Initially, FVL has been associated in the literature with few scenarios: announcing a macular hole (MH), in multiple evanescent white dot syndrome (MEWDS), during macular hemorrhage and as a sequel to resorbed macular edema. [1–3].

In another study by Tsujikawa et al. macular edema resolution associated with retinal vein occlusion showed FVL in 13.19% (12/91) of the examined eyes. [7]

Ishibashi et al. suggested a potential prognostic value for FVL, noting that all cases with parafoveal epiretinal membrane (ERM) that developed to secondary MH after pars plana vitrectomy (PPV) for rhegmatogenous retinal detachment (RRD) exhibited FVL [3].

FVL was described in a range of conditions, including age-related macular degeneration (AMD), adult-onset foveomacular dystrophy, MEWDS, pachychoroid pigment epitheliopathy, and fundus flavimaculatus. [4, 5] These findings suggest that FVL can manifest in a broader spectrum of retinal diseases than previously understood, necessitating a more inclusive examination of its etiologies and clinical significance.

In this observational study, we intend to extend and review the differential diagnosis of FVL.

## Patients and methods

This observational case series study was conducted in the retina unit of Shaare Zedek Ophthalmology Department, a tertiary eye care referral center.

It was approved by the Institutional Ethics Committee (approval number 0248–21 SZMC(.

The sole inclusion criterion was the demonstration of FVL on SD-OCT structural images. FVL was defined as any vertical hyperreflective line of more than 40 microns at the fovea, at any level from the retinal nerve fiber layer to the pigment epithelium.

Patients whose picture quality was too low to enable unequivocal interpretation study and those who had undergone retinal surgery were excluded from our research.

We gathered patients from those who presented at the medical retina consultation (JH) with FVL from March to September 2021. We reviewed their underlying disease, diagnosed by clinical exam and ancillary tests.

Macular spectral-domain OCT scans were performed on all studied eyes using the Heidelberg Spectralis device (software version 6.9.4.0; Heidelberg Engineering, Germany) following the same protocol consisting of a dense macular scan, high-speed mode, > 15 images averaged, scan angle 20 degrees, 5.9 × 5.9 mm, X scaling 11.47 µm/pixel, and Z scaling 3.87µm/pixel. When available, we reviewed other OCT images taken within 2 2-year period before or after the study OCT image.

When analysis of volumetric OCT pictures was insufficient to establish the proper diagnosis, the patients underwent multimodal imaging, including fluorescein angiography, OCT-angiography, enhanced deep imaging OCT and autofluorescence.

Integrating all investigations, including medical history, fundus examination, OCT, and optional extra imaging, we retrieved seven diagnoses associated with FVL.

## Results

The study included ten eyes of 10 patients that displayed FVL in SD-OCT. No patient was excluded due to insufficient imaging quality or previous retinal surgery. The mean age at presentation was 69 years (SD ± 18.37, range 25–90). FVL were unilateral in all the patients.

The presence of FVL on SD-OCT was retrieved in the following settings: tractional disorders of the vitreoretinal interface, accompanying fluid resorption, inflammation, macular telangiectasia, AMD, diabetic retinopathy, and scarring.

Patients' clinical characteristics and associated morphological and retinal diagnoses are presented in Table 1.

**Table 1:** Patients' characteristics

Diagnosis	patient n	Gender	Age	Eye	Medical history	BCVA in the examination date Snellen (LogMar)	Deepest involved layer	Intraretinal fluids (Yes/No)	Duration of FU	Resolution or improvement in last FU	BCVA at end of FU Snellen (LogMar)
Inflammation	A	F	25	LE	Pregnant/Myopia	20/30 (0.18)	RPE	No	1	Yes	20/25 (0.1)
Mechanical	B	M	85	LE	HTN/Asthma/Cardiomyopathy /Hyperlipidemia/IHD	20/40 (0.3)	ONL	No	19	NO	20/150 (0.88)
Resorption of fluids	C	M	71	LE	Chron's/HTN/DM/ Hypothyroidism/Anemia	20/30 (0.18)	RPE	No	22	Yes	20/20 (0)
	D	F	79	LE	Hormonal Substitution	20/30 (0.18)	EZ	No	25	Yes	20/40 (0.3)
	H	F	66	RE	DM/Nephropathy/HTN/ Dyslipidemia/Hypothyroidism	20/20 (0)	ELM	Yes	21	Yes	20/25 (0.1)
	I	M	69	LE	DM/Dyslipidemia/HTN/ CHF/COPD/BPH	20/30 (0.18)	RPE	Yes	19	Yes	20/50 (0.4)
Macular telangiectasia	E	F	58	LE	Churg Strauss Vasculitis/HTN /CVA/Ulcerative Colitis/Asthma	20/30 (0.18)	ONL	Yes	26	Yes	20/40 (0.3)
AMD	F	M	81	RE	HTN/GERD/IHD/Cataract Surgery	20/150 (0.88)	ELM	No	21	No	20/30 (0.18)
Diabetic retinopathy—drill	G	M	64	RE	DM/HTN/IHD/Dyslipidemia/ Prostate Cancer/Hepatitis B	20/50 (0.4)	ELM	Yes	7	Yes	20/30 (0.18)
Scar	J	M	90	RE	HTN/GERD/Dementia	20/2510 (2.1)	RPE	No	19	No	20/280 (1.15)
Average ± SD (range)			69 ± 18.37 (25,90)			0.46 ± 0.62(0,2.1)			18 ± 7.89(1,26)		0.36 ± 0.37(0.1,1.15)

BCVA = best corrected visual acuity; FU = follow up; F = female; M = male; RPE = retinal pigment epithelium; HTN = hypertension; IHD = ischemic heart disease; ONL = outer nuclear layer; LE = left; RE = right; EZ = ellipsoid zone; DM = diabetes Mellitus; ELM = external limiting membrane; CHF = congestive heart failure; COPD = chronic obstructive pulmonary disease; BPH = benign prostatic hyperplasia; CVA = cerebral vascular accident; GERD = gastroesophageal reflux disease; SD = standard deviation

**Fig. 1:**
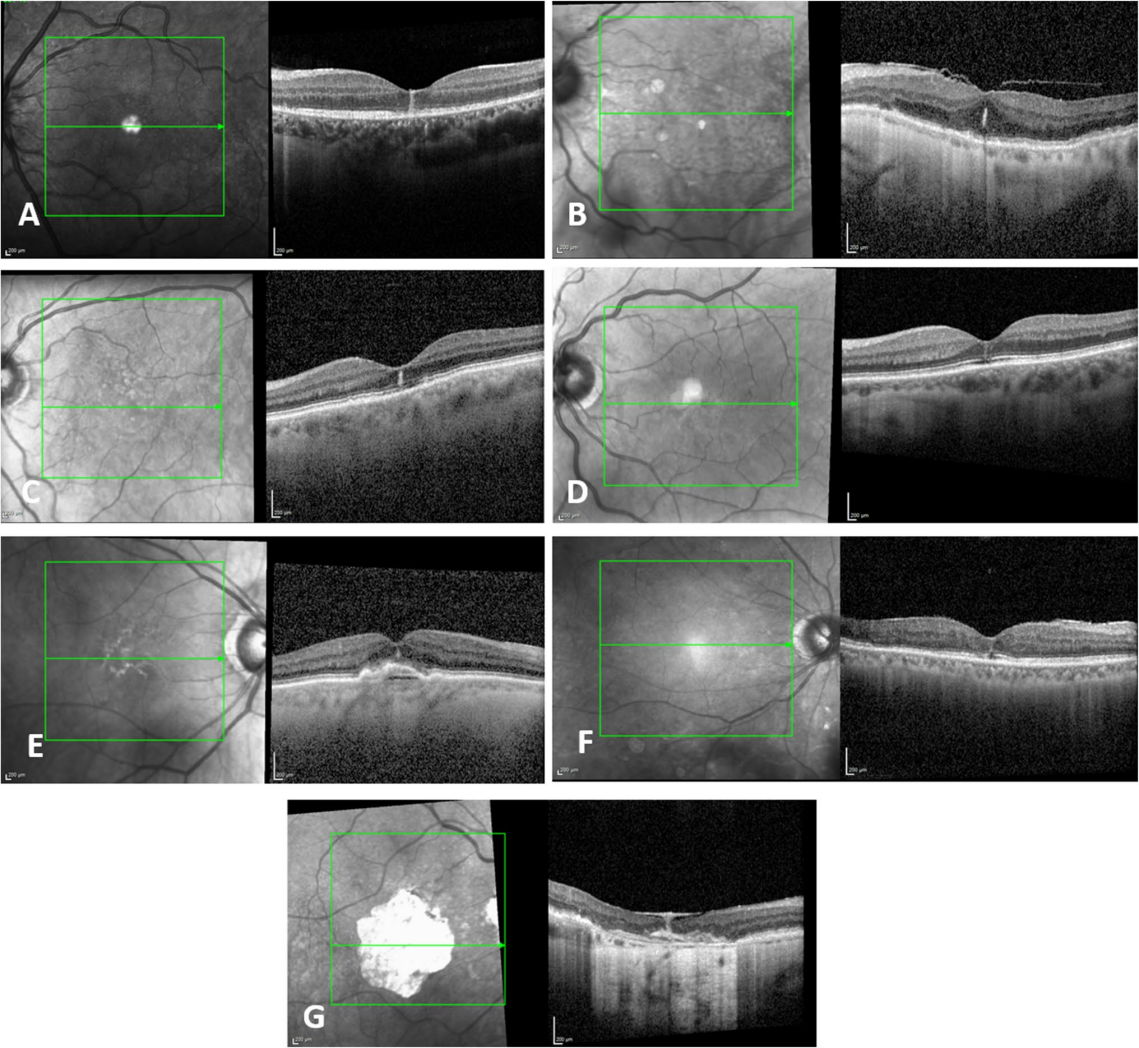
Examples of FVL as revealed by SD-OCT in different clinical settings: A: Inflammation; B: Mechanical; C: Resorption of fluids;D: Macular telangiectasia; E: AMD; F: Diabetic retinopathy; G: Scar

Figure 1 exhibits examples of FVL for each diagnosis

## Discussion

In this study, we describe FVL as a specific morphological finding on structural SD-OCT and discuss its differential diagnosis in a case series of 10 eyes of 10 patients.

The study findings revealed that FVL could be identified through SD-OCT in various settings. FVL was not only associated with tractional disorders of the vitreoretinal interface or accompanying fluid resorption but also in the context of inflammation, macular telangiectasia, AMD, diabetic retinopathy, and scarring. No FVL-specific pattern, isolated from broader morphological elements or the clinical context, was found to characterize any of those conditions.

Analyzis of those specific cases, in their proper clinical context, enables us to describe various conditions in which FVL may appear.

### Mechanical

In their landmark study of hyperreflective stress lines and macular holes, Scharf et al. described FVL as an early OCT marker for FTMH development. [2] They retrieved FVL in 6 over 12 eyes (50%) that underwent OCT before the development of FTMH and, post-surgically, in 26 over 51 eyes (51%) in which pars plana vitrectomy was performed with anatomical success defined as closure. They also analyzed 88 eyes with lamellar macular holes and noticed FVL in 22 (25%). The authors postulated that in this context, FVL might be a sign of central foveal dehiscence owing to disruption of the Muller cell cone. The common denominator in all cohorts may be the presence of a naturally existing cleavage plane or seam in the central fovea that is either being pulled apart or pulled together in these three clinical situations. [2]

### Resorption of intraretinal fluid

Whatever the etiology of macular edema, its resorption may be accompanied and followed by FVL.

Two mechanisms may explain the appearance of FVL in this setting: on the one hand, the deposition of lipidic and proteinaceous material similar to hard exudates, favored by the predisposing existence of relatively decreased resistance at the very center of the fovea; on the other hand, the activation of cellular inflammatory processes that include activation of the local microglia and recruitment of macrophages. [8]

Hasegawa et al. reviewed 59 eyes with resolved macular edema related to branch retinal vein occlusion and found FVL in 21 (36%). [1] They noticed that FVL were associated with a disrupted external limiting membrane before the resolution of macular edema and, after the resolution of macular edema, with an interruption of the ellipsoid zone, therefore making FVL a marker of damage to the photoreceptors.

To our knowledge, FVL has not yet been systematically studied in diabetic macular edema, the most common cause of macular edema. However, subretinal fluid and foveal plaque [9] are well-known markers of chronicity and severity in diabetic macular edema. FVL might be closely related to the pathophysiology of those conditions. In retinal vein occlusion, for instance, FVL has been described as a track of the passage through which intraretinal fluid within the cystoid spaces flows into the subretinal space. [9]

### Diabetic retinopathy

Besides just described OCT observable phenomena related to the resorption of intraretinal fluid, inflammatory mechanisms can result in FVL.

Bolz et al. described hyper-reflective foci in diabetic retinopathy. Those are punctate lesions scattered throughout the retina. [10] Whatever their exact nature, still controversial but intimately associated with inflammation (activated microglial cells, degenerated photoreceptor cells, anteriorly migrated retinal pigment epithelium (RPE), vascular components), those should be differentiated from hard exudates. [11] However, in the fovea, they could combine with those later and adopt different patterns [12], including vertically linear, to display FVL on OCT.

### Macular telangiectasia

MacTel 2 can be divided into non-proliferative and proliferative stages, with neovascularization in the advanced disease. [13] In the non-proliferative stage of MacTel 2, a hyperreflective middle retinal layer from capillary leakage can be noted on imaging. Findings of irregular fovea and hyperreflective RPE clumps are common in the advanced stage of MacTel 2 with poor vision.Combined with inner and outer retinal hyporeflective cavities accentuating the contrast, retinal crystals seen as hyperreflective spots in the superficial layers of the retina and outward turning of the inner retinal layers, those can result in the appearance of FVL, in possible further association with the hyperreflective elements related to the neovascular complication. [14]

### Inflammation

Hyperreflective dots have been described in several forms of intraocular inflammation. We have already mentioned their common presence in diabetic macular edema. They have also been observed in infectious[15] and non-infectious uveitis[16], after uncomplicated cataract surgery[17], in neurological conditions such as multiple sclerosis [18] as a marker of inflammation in retinitis pigmentosa[19] as well as in von Hippel-Lindau disease[20] or with Covid-19. [20] It should be noticed that hyperreflectivity displayed on averaged volumetric scans can exhibit a linear pattern, appearing as FVL. Cohen’s team hypothesized that FVL could correspond to a previously unrecognized reaction to various photoreceptor, Müller cell, and/or RPE damage [4, 6].

### AMD

Deep hyperreflective dots were described early in OCT studies of age-related macular degeneration. [21]

Hyperreflective dots were generally believed to represent anteriorly migrating RPE cells and possible disaggregated photoreceptors. [22] It is currently admitted that those foci may also represent microglia migrating from the inner to the outer retinal layers engorged by lipid droplets or cholesterol. [23] Balaratnasingam et al., studying the RPE behavior in AMD by multimodal ex vivo imaging including SD-OCT and high-resolution histology, defined several histology-OCT correlations in D-PED: small and large hyperreflective intraretinal foci represent fully pigmented and nucleated RPE cells that migrate anteriorly either singly or in groups; hyperreflectivity internal to the RPE-BL band resembling vitelliform lesions represent subretinal plaques of RPE organelles mixed with outer segment debris and sometimes also RPE cell bodies; punctate hyperreflective foci in the D-PED interior represent refractile material among the lipid pools. Microglial activation is mainly related to neovascular disease. [23] Those hyperreflective structures have variable morphological characteristics such as size, migration, and clumping. They can eventually adopt a linear pattern. [23]

Subretinal hyper-reflective material (SHRM) is a morphological feature seen on OCT as hyper-reflective material located external to the retina and internal to the RPE. SHRM may be attributed to a heterogeneous group of lesions, including gray exudative fluid, hemorrhage, vitelliform material, or type 2 CNV. [24] It may represent many elements, from neovascular tissue to fibrin, blood, and lipids.

Hyperreflective dots located above the external limiting membrane, often co-localized with a drusenoid pigment epithelial detachment, can also represent a nascent type 3 macular neovascularization, often characterized by vertical growth. [25]

### Scar

Fibrosis, appearing as a hyperreflective structure, is the end-stage complication of many destructive chorioretinal pathologic processes, AMD being the most frequent. Alone or in combination with other disease markers, it can adopt a vertical pattern, displaying the features of FVL on OCT [26].

There are obvious limitations to our study. Its observational design, retrospective nature, and relatively small sample size may limit the findings' generalizability. We cannot pretend to dress an exhaustive list of etiologies for FVL but enhance awareness of these unique features revealed by SD-OCT. Further research with larger cohorts is warranted to validate these observations, establish the prevalence of FVL in different etiologies, and describe its long-term behavior and prognostic significance. Histologic studies would be required to determine the cellular correlation of this reflectivity pattern in selected conditions. It is essential to acknowledge that patients can present with FVL and that recognizing it, with the help of other orienting clues, is capital in determining the correct diagnoses and offering the most accurate treatment.

In conclusion, in this study, we describe and identify FVL as a specific morphological finding on SD-OCT in patients with various ophthalmic conditions. The comprehensive discussion of the differential diagnoses associated with FVL aids ophthalmologists in making accurate diagnoses and implementing appropriate treatment strategies. Further studies with larger sample sizes are necessary to evaluate more etiologies for FVL and the clinical implication of this yet underestimated phenomenon.

## Declarations

This article does not contain any studies with human participants or animals performed by any of the authors.
